# Erdheim-chester disease revealed by diabetes insipidus

**DOI:** 10.11604/pamj.2019.33.293.19194

**Published:** 2019-08-13

**Authors:** Bilel Ben Amor, Hanene Sayadi, Manel Jemel, Houcem Mrabet, Rym Hadhri, Tensim Slim, Rym Klii, Ines Khochtali

**Affiliations:** 1 Fattouma Bourguiba University Hospital, Department of Endocrinology, Monastir, Tunisia; 2 National Institute of Nutrition, Department of Endocrinology Tunis Tunisia; 3 Fattouma Bourguiba University Hospital, Department of Anatomopathology, Monastir, Tunisia

**Keywords:** Erdheim-chester disease, diabetes insipidus, interferon alpha

## Abstract

Erdheim-Chester disease (ECD) is a very rare and aggressive form of non-Langerhans histiocytosis with unclear pathogenesis. Because of the heterogeneity of clinical presentation, diagnosis is often challenging and delayed. Currently, Interferon alpha is the first line treatment that is associated with a better survival. The prognosis is relatively poor, especially in case of neurological and cardiovascular involvement. Herein, we report the case of a 64-year-old Tunisian female patient presenting an aggressive form of ECD revealed by diabetes insipidus and cerebellar ataxia with a diagnosis delay of 4 years. The assessment of disease extent had also shown associated asymptomatic cardiac and bone involvement. Pegylated Interferon alpha was started at high dose allowing disease stabilization. This case illustrates that physicians should be aware of the heterogeneous manifestations of ECD in order to insure an early diagnosis and treatment. Long-term and regular follow-up is crucial because of the risk of disease progression.

## Introduction

Erdheim-Chester disease (ECD) is an aggressive form of non-Langerhans histiocytosis, characterized by multi-organ infiltration by CD 68+, CD1a- foamy histiocytes [1]. Since its first description in 1930, only 500 cases have been reported. This makes ECD a very rare disease with an unclear pathogenesis [1,2]. Patients with ECD have a varied clinical presentation depending on the distribution of lesions [1,3]. Some symptoms, appearing in varying combination, are suggestive of the diagnosis, which is confirmed by histology and immunohistochemistry studies. Neurological manifestations are common, and central nervous system (CNS) involvement is associated with poor prognosis [1,4]. However, endocrine disorders are rare, among them, diabetes insipidus is a characteristic manifestation of the disease [1]. The first line treatment is based on Interferon 45; (IFN-45) that significantly improves survival [4]. The prognosis is mainly related to the severity and extent of neurological and cardiovascular involvement [1]. In this paper, we report a case of ECD revealed by cerebellar ataxia associated with polyuria- polydipsia syndrome and we discuss the particularities of the clinical presentation and evolution of our patient.

## Patient and observation

**Figure 1:**
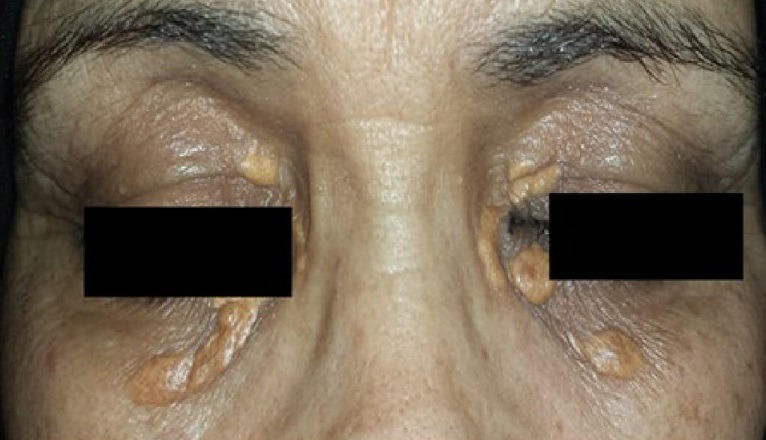
Eyelid xanthelasmas

**Figure 2:**
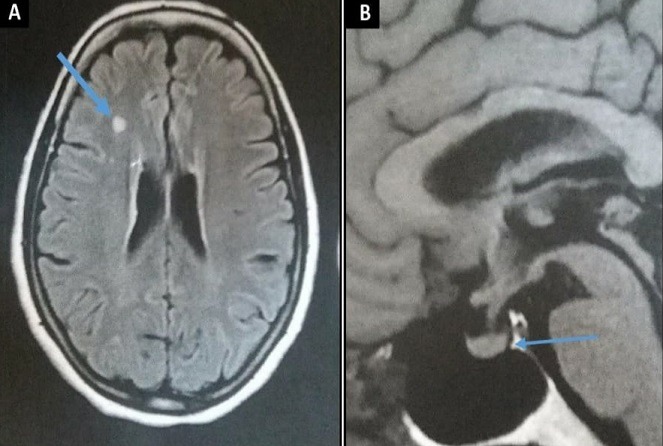
A) brain and Hypothalamo-pituitary MRI showing white matter nodular hyperintensities; B) the loss of hyperintense signal on sagittal T1 weighted image

**Figure 3:**
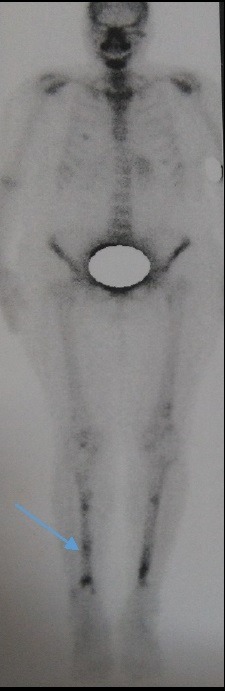
Tc99m-methyl-diphosphonate scintigraphy showing symmetric uptake of the radiotracer in the tibial diaphysis and metaphysis regions

**Figure 4:**
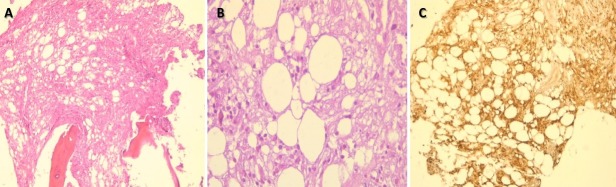
A) bone infiltration by foamy histiocytes with fibrosis; B) hematoxylin eosin X 100; C) hematoxylin eosin X 400) and positive immunostaining of histiocytes for CD68 (x100)

It consists in a 64-year-old female patient presented with persistent polyuria and polydipsia during the last 4 years with difficulties to walk. She had no history of diabetes mellitus, cranial trauma or surgery. The patient had consulted several times before referring her to the internal medicine and endocrinology department for polyuria-polydipsia syndrome investigation. Physical examination showed eyelid xanthelasmas (Figure 1) and cerebellar ataxia. Diabetes mellitus was ruled out on routine workup and assessment of anterior pituitary functions was normal. After a water deprivation test combined with Desmopressin administration, central diabetes insipidus was confirmed. Hypothalamo-pituitary magnetic resonance imaging (MRI) showed no pituitary stalk abnormalities but the loss of hyperintense signal on sagittal T1 weighted imaging. It also showed white matter nodular hyper intensities on fluid attenuated inversion recovery (FLAIR) brain MRI (Figure 2). Because of this association (diabetes insipidus, cerebellar ataxia, and xanthelasmas), diagnosis of ECD was suspected. Bone involvement was investigated by Tc^99m^ methyl-diphosphonate (MDP) scintigraphy that showed symmetric uptake in both tibias, on the left radius and on the left femur (Figure 3). Proximal tibia bone biopsy was then performed. Histopathological findings showed bone infiltration by foamy histiocytes with fibrosis. Immuno-histochemical staining was positive for CD68 and negative for CD1a (Figure 4). Once the diagnosis was confirmed, the involvement of other organs was assessed by transesophageal echocardiography that showed a left ventricular hypertrophy and pericardial effusion, while thoracoabdominal tomodensitometry showed pericardial thickening with minimal pericardial effusion. The aorta, adrenal glands and kidneys were normal. There were no pulmonary abnormalities or retroperitoneal infiltration. The patient was initially treated with Prednisone (1mg/kg/day). Three months later, she developed corticosteroids-induced diabetes that responded well to treatment with Metformin and Insulin NPH. Corticosteroids was gradually stopped and pegylated IFN-α was initiated at the dose of 180 μg/ week. After 9 months of treatment, patient was clinically stable and there is no disease progression.

## Discussion

ECD is a very rare non-Langerhans histiocytosis. In the 2016 World Health Organization classification of lymphoid neoplasms, ECD has been added to histiocytic and dendritic cell neoplasms [5]. This disease affects typically adults between their 5^th^ to 7^th^ decade of life with a slight male predominance (male to female ratio: 1.5) [1,6]. Because of its rarity and its variable clinical presentation, the correct diagnosis is often delayed with median diagnostic delay of 1 year (range, 0-34) [2]. In recent years, a better physician's awareness has led to a rapid increase in the number of new cases and the decrease in the mean delay of diagnosis [1]. The 4-year delay in our case may be explained by the insidious apparition of the polyuria and the absence of alerting symptoms like bone pain. In the case of our patient, the association of cerebellar ataxia, diabetes insipidus and xanthelasmas was suggestive of ECD. In fact, ECD is an infiltrative disease. Hence, it can affect any organ and system. CNS involvement is present in up to 50% of patients. Non-specific, neurological manifestations can evolve several years before suspecting diagnosis of ECD. The most frequent manifestations were: pyramidal syndrome (45%), cerebellar syndrome (41%), neuro-psychiatric symptoms/cognitive impairment (21%) and seizure (12%) [7]. Brain MRI should be systematically performed in all new cases of ECD. On MRI, 3 forms of SNC lesion were described (infiltrative, meningeal and composite forms) [1,7]. Hypothalamic and pituitary infiltration led to endocrine manifestations. Hypopituitarism is rare, reported in 6% of cases [6]. Central diabetes insipidus is the most common and the most characteristic among endocrine abnormalities. It can be seen in 29 to 48% of cases [6,7]. Generally it appears early in the history of the disease [8]. Adrenal infiltration was also described, but insufficiency is extremely rare [1]. Others endocrine manifestations include hyperprolactinemia and deficiency of insulin-like growth factor 1.

The third evocative symptom in the case of our patient was eyelid xanthelasmas. This sign is present in 28% of patients and can be associated with xanthomas and mucosal infiltration of the genital area seen in 1% of cases [3,6]. Skin abnormalities are more common in older patients [6]. In the literature, bone pain described by patients in 50% of cases, was the most frequent symptom leading to the diagnosis. However, skeletal involvement was reported in the majority of ECD patients [96%] [8]. As in the case of our patient, bone involvement was asymptomatic and it was demonstrated by Tc^99m^ MDP scintigraphy that showed symmetric uptake of the long bones of the legs which is very specific of ECD [1,3]. Another severe and prevalent manifestation of ECD, cardiac involvement was reported in more than 70% of patients [3]. All heart tunics can be affected. Pericardial infiltration and/or effusion is the most common heart lesion (42% of cases). However, tamponade was rarely observed [1,3]. Cardiac imaging can also show myocardial hypertrophy with pseudo-tumoral infiltration of the right atrium and infiltration of the auriculo-ventricular sulcus [3,8]. Valvulopathy and pericoronarial infiltration leading to myocardial infraction was reported in some cases [3,4]. The circumferential infiltration of the aorta, described as “coated aorta” is nearly pathognomonic for ECD and it was seen in 38% of cases but not in the case of our patient [1,3]. This infiltration can extend to the aortic branches, generally without severe clinical consequences except renal artery involvement, leading to nephrovascular hypertension [3]. Many other organs are affected in ECD, among them pulmonary involvement has been overlooked because it is rarely symptomatic [1,9]. Its impact on the prognosis is still controversial, as well [3]. Retroperitoneal infiltration is also frequent (30% of cases) [1]. This infiltration is characterized by a typical radiological sign “hairy kidney”, but sometimes it leads to serious complications such as hydronephrosis and renal failure [1,3]. Retro-orbital soft tissue infiltration and proptosis can be seen in 25% of patients. Other rare localizations of the disease were reported such as thyroid, testes, lymph node and breast [3].

Given the heterogeneous clinical presentation and the overlap of several signs with other systemic diseases, the diagnosis is challenging. Haroche J *et al*. [3] proposed the following two criteria. The first and major criterion is typical histology, revealing CD68+, CD163+, CD1a- and S100- or weakly positive-foamy histiocytes organized into polymorphic xanthogranulomas and accompanied by fibrosis. The second criterion is a distinctive bone involvement characterized by symmetric cortical osteosclerosis of the diaphyseal and metaphyseal regions in the long bone with increased labeling on the Tc^99m^ MDP scintigraphy. Prior to 2005, the treatment of ECD was based on corticosteroids and cytotoxic chemotherapy. Presently, many other therapeutic options are available with variable efficiency, depending on the extent and severity of the disease [10]. Among these options, IFN-α has the largest amount of supporting evidence as a first line treatment for ECD [10]. In severe forms with CNS and cardiovascular involvement, IFN-α is prescribed at high dose. The optimal duration is still undefined but long term (> 3 years) treatment is associated with a greater chance of stabilization or improvement in high risk ECD [10]. This was noted in the case of our patient, who after 9 months of treatment, has a stable disease. In patients with refractory and severe ECD, harbouring BRAF^V600E^ mutation, vemurafenib should be considered [10]. SNC involvement has a negative impact on patient autonomy and quality of life and it is an independent predictor factor of death [4]. Coexisting cardiovascular involvement is associated with worse prognosis considering that 60% of patients' mortality is due to cardiac complications [8]. Yet, the response to IFN-45; may improve the patients' survival [4].

## Conclusion

Our case illustrates the difficulty of an early diagnosis of ECD even when suggestive manifestations are present. For this reason, physicians should consider this rare and severe entity in the differential diagnosis of many systemic conditions. In the aggressive forms of ECD, early onset of a high dose of IFN-α can control the disease and improve quality of life. Long-term monitoring and follow-up are crucial because of the risk of disease progression.

## Competing interests

The authors declare no competing interests.

## References

[1] Campochiaro C, Tomelleri A, Cavalli G, Berti A, Dagna L (2015). Erdheim-Chester disease. Eur J Intern Med.

[2] Cavalli G, Guglielmi B, Berti A, Campochiaro C, Sabbadini MG, Dagna L (2013). The multifaceted clinical presentations and manifestations of Erdheim-Chester disease: comprehensive review of the literature and of 10 new cases. Ann Rheum Dis.

[3] Haroche J, Arnaud L, Cohen-Aubart F, Hervier B, Charlotte F, Emile JF (2014). Erdheim-Chester disease. Curr Rheumatol Rep.

[4] Arnaud L, Hervier B, Néel A, Hamidou MA, Kahn J-E, Wechsler B (2011). CNS involvement and treatment with interferon-α are independent prognostic factors in Erdheim-Chester disease: a multicenter survival analysis of 53 patients. Blood.

[5] Swerdlow SH, Campo E, Pileri SA, Harris NL, Stein H, Siebert R (2016). The 2016 revision of the World Health Organization classification of lymphoid neoplasms. Blood.

[6] Ci7ves M, Simone V, Rizzo FM, Dicuonzo F, Cristallo Lacalamita M, Ingravallo G (2015). Erdheim-Chester disease: A systematic review. Crit Rev Oncol Hematol.

[7] Lachenal F, Cotton F, Desmurs-Clavel H, Haroche J, Taillia H, Magy N (2006). Neurological manifestations and neuroradiological presentation of Erdheim-Chester disease: report of 6 cases and systematic review of the literature. J Neurol.

[8] Mazor RD, Manevich-Mazor M, Shoenfeld Y (2013). Erdheim-Chester Disease: a comprehensive review of the literature. Orphanet J Rare Dis.

[9] Arnaud L, Pierre I, Beigelman-Aubry C, Capron F, Brun A-L, Rigolet A (2010). Pulmonary involvement in Erdheim-Chester disease: a single-center study of thirty-four patients and a review of the literature. Arthritis Rheum.

[10] Diamond EL, Dagna L, Hyman DM, Cavalli G, Janku F, Estrada-Veras J (2014). Consensus guidelines for the diagnosis and clinical management of Erdheim-Chester disease. Blood.

